# Dendritic Cells as Sensors, Mediators, and Regulators of Ischemic Injury

**DOI:** 10.3389/fimmu.2019.02418

**Published:** 2019-10-15

**Authors:** Helong Dai, Angus W. Thomson, Natasha M. Rogers

**Affiliations:** ^1^Department of Urological Organ Transplantation, The Second Xiangya Hospital of Central South University, Changsha, China; ^2^Clinical Research Center for Organ Transplantation of Hunan Province, Changsha, China; ^3^Department of Surgery, Thomas E. Starzl Transplantation Institute, University of Pittsburgh School of Medicine, Pittsburgh, PA, United States; ^4^Department of Immunology, University of Pittsburgh School of Medicine, Pittsburgh, PA, United States; ^5^Center for Transplant and Renal Research, Westmead Institute for Medical Research, Westmead, NSW, Australia; ^6^Renal Division, Westmead Hospital, Westmead, NSW, Australia; ^7^Westmead Clinical School, University of Sydney, Camperdown, NSW, Australia

**Keywords:** dendritic cells, ischemic injury, kidney, liver, heart

## Abstract

Dendritic cells (DCs) are highly specialized, bone marrow (BM)-derived antigen-processing and -presenting cells crucial to the induction, integration and regulation of innate, and adaptive immunity. They are stimulated by damage-associated molecular patterns (DAMPS) via pattern recognition receptors to promote inflammation and initiate immune responses. In addition to residing within the parenchyma of all organs as part of the heterogeneous mononuclear phagocyte system, DCs are an abundant component of the inflammatory cell infiltrate that appears in response to ischemia reperfusion injury (IRI). They can play disparate roles in the pathogenesis of IRI since their selective depletion has been found to be protective, deleterious, or of no benefit in mouse models of IRI. In addition, administration of DC generated and manipulated *ex vivo* can protect organs from IRI by suppressing inflammatory cytokine production, limiting the capacity of DCs to activate NKT cells, or enhancing regulatory T cell function. Few studies however have investigated specific signal transduction mechanisms underlying DC function and how these affect IRI. Here, we address current knowledge of the role of DCs in regulation of IRI, current gaps in understanding and prospects for innovative therapeutic intervention at the biological and pharmacological levels.

## An Introduction to Dendritic Cells (DCs)

DCs comprise a heterogeneous population of uniquely well-equipped, bone marrow-derived innate immune cells. They are distributed ubiquitously throughout the body and play an important homeostatic and anti-infectious sentinel role. DCs are highly efficient, antigen (Ag)-acquiring, -processing, and presenting cells, that perform crucial roles in the instigation and regulation of acute and chronic inflammatory responses. While they promote self-tolerance in the healthy steady-state and can be targeted by microbes and tumors to evade immunity, DCs integrate innate and adaptive immunity effectively to combat infection and can also be exploited as anti-cancer vaccines. During autoimmunity and transplant rejection, DCs instigate deleterious immune responses that cause disease; on the other hand, they can be harnessed to silence these conditions using novel targeting and adoptive cell therapy approaches. In the context of ischemic tissue injury that adversely affects short- and long-term transplant outcome, DCs appear to play diverse roles in regulation of the inflammatory response.

## DC Subsets—Phenotype and Function

Distinct subsets of DCs, including myeloid/conventional DCs (cDCs) and type-I IFN-producing plasmacytoid DCs (pDCs) have been described extensively elsewhere (1–3) and are summarized in Table 1. Classical DCs, but not monocyte-derived antigen (Ag)-presenting cells (APCs), are critical for central and peripheral regulatory T cell (Treg) induction and the development of tolerance (4) as well as shaping effector T cell responses to Ag. Beyond the classical characterization of DCs, new phenotypic and functional subsets of DCs continue to emerge (5, 6). Moreover, the discovery of new lineage markers and introduction of innovative imaging technologies (including use of reporter mice) have helped to distinguish classical DC subsets from other myeloid cells, particularly macrophages, in tissues such as the kidney (7).

**Table 1:** DC subsets in mouse and human: phenotype, localization, and function.

**Human DC subsets**	**Phenotype**	**Location**	**Function**
Plasmacytoid DC	CD123 CD303/BDCA-2 CD304/BDCA-4	Blood, tonsil, non-lymphoid tissues	Production of type I and III IFN
Myeloid cDC1	CD141/BDCA-3	Blood, tissues, and lymphoid organs	Present Ag to CD8+T cells and produce type III IFN
Myeloid cDC2	CD1c/BDCA-1 CD2 CD11c CD11b	Blood, tissues and lymphoid organs	Activate Th1/Th2/Th17 and CD8^+^T cells
Langerhans cells	CD207 CD1a E-Cadherin	Skin (epidermis)	Transfer Ag to afferent lymphatics, stimulate CD8^+^T cells
Mo-DC	CD11c CD1c/BDCA-1 CD1a	*Ex vivo*-generated	
**Murine DC subsets**			
Plasmacytoid DC	CD11c^int^ CD11b^−^ CD8^−^B220^+^Gr-1^+^	Lymphatic and non-lymphoid tissue	IFN-α production
CD8^+^ cDC	CD11c^+^ CD11b^−^CD8^+^ CD24^+^ MR^+^	Lymphatic and non-lymphoid tissue	Activate CD4^+^ and CD8^+^ T cells, Ag presentation
CD8^−^ cDC	CD11c^+^ CD11b^+^CD8^−^ CD24^−^	All tissues	Activate CD4^+^ T cells, transport Ag to LN
Langerhans cell	CD11c^+^ CD11b^+^ CD8^−^Langerin^+^ CD1a^+^	Skin (epidermis)	Transport Ag from skin to LN

*CD, cluster of differentiation; cDC, conventional DC; DC, dendritic cell; Mo-DC, monocyte-derived DC; pDC, plasmacytoid DC; LN, lymph nodes; MR, mannose receptor; IFN, interferon*.

According to their maturation and functional status, DCs can be divided into immature, mature, and regulatory populations. Regulatory DCs (DCregs) have been extensively investigated in transplantation (8–11) and autoimmune disease (12, 13), ranging from pre-clinical models to pilot human clinical trials. Infusion of donor-derived DCregs prior to transplantation has been shown to prolong kidney allograft survival and inhibit donor-reactive CD8^+^ memory T cell responses in non-human primates (14, 15). First-in-human phase I/II clinical trials of adoptive DCreg therapy in living donor renal and liver transplantation have recently been instigated (8, 16).

## Ischemia-Reperfusion Injury (IRI)

IRI is a common clinical condition triggered by various physiological derangements (sepsis, cardiogenic shock, vascular surgery, organ retrieval for transplantation). Its pathogenesis has been comprehensively described elsewhere (17–21) but is essentially characterized by endothelial dysfunction, reactive oxygen species (ROS) production, secretion of pro-inflammatory mediators, and recruitment of inflammatory cells which exacerbate/perpetuate tissue injury.

## Inflammatory Cells Characterize IRI

**Figure 1:**
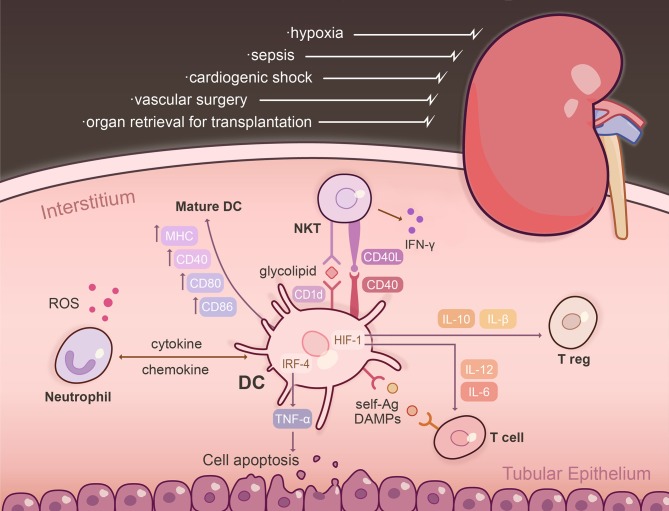
The DC interactome following renal ischemia reperfusion injury (IRI). IRI is a common clinical condition triggered by various physiological derangements including sepsis, cardiogenic shock, vascular surgery, and organ retrieval for transplantation. Following injury, resident, and influxing DCs become activated within the kidney parenchyma and are the dominant TNFα-producing cells. The effect of TNF-α is dependent on the transcription factor IRF-4, promoting renal tubular epithelial cell apoptosis, glomerular endothelial damage, and fibrin deposition. Post-IRI, intra-renal DCs upregulate markers that designate them as mature APCs, including MHC, CD80, CD86, CD40, and CD1d. Activation of NKT cells via CD40 initiates IFN-g to amplify the innate immune response. Renal DCs are capable of presenting self-Ag to a variety of T cells in the context of IRI: CD11c^+^ DCs cross-present Ag to CD8^+^ T cells and glycolipids are presented to NKT cells via CD1d. Exposure of DCs to hypoxia/reperfusion augments production of IL-12 and IL-6, and an inflammatory T cell phenotype through HIF-1α transcriptional regulation. Absence of DC-specific HIF-1α limits expression of IL-10 and TGF-β, which are potent inducers of Tregs. DCs intercommunicate with neutrophils and NKT cells to enhance tissue injury through chemokine/cytokine secretion, as well as cell-based contact. Neutrophils release ROS and proteolytic enzymes, and NK1.1^+^CD161^+^ NKT cells elaborate pro-inflammatory cytokines and are the predominant early inflammatory cells that impair organ function within hours of IRI. Ag, antigen; DAMPs, damage-associated molecular patterns; HIF-1, hypoxia-inducible factor-1; IRF-4, interferon regulatory factor-4; ROS, reactive oxygen species.

Inflammatory cell infiltration after IRI is rapid, peaking 24 h following reperfusion (22). Gr-1^+^ neutrophils, which release ROS and proteolytic enzymes, and NK1.1^+^CD161^+^ NKT cells (CD56^+^ in humans) which elaborate pro-inflammatory cytokines, are the predominant early inflammatory cells that impair organ function within hours of IRI (23–26). However, neutrophil-depleted animals are not protected following ischemic insult to the kidney (27, 28). Neutrophils, NKT cells and DCs intercommunicate to enhance tissue injury through chemokine/cytokine secretion, as well as cell-cell contact (29, 30). These interactions are depicted in Figure 1 in the context of the early period following renal IRI. While DCs are thought to be crucial to the pathogenesis of IRI, their role in kidney IRI remains unclear, since depletion may be protective (22), deleterious (31), or of no benefit (32). *In situ* targeting of DCs with the vitamin D analog paricalcitol can induce intrahepatic tolerogenic DCs and alleviate CD4^+^ T cell responses to attenuate hepatocellular damage (33). In contrast, pDC-released type I IFN promotes tissue injury through induction of hepatocyte IFN regulatory factor-1 (IRF-1) to induce apoptosis (34).

Macrophages, NK cells and adaptive immune cells, including T and B cells, infiltrate injured tissues hours after reperfusion (22). Macrophages have been considered to polarize into M1 (classical) and M2 (alternatively-activated) subsets with pro- or anti-inflammatory function, respectively, although recent reassessment suggests a broader functional repertoire for these cells (35). Heme oxygenase 1 (HO-1) negatively regulates M1 polarization and hepatocellular damage in both mouse liver IRI and human liver transplant biopsies (36). Further evidence shows that HO-1 regulates macrophage activation through the Sirtuin/p53 signaling pathway to drive hepatic death during liver IRI (37). Deletion or inhibition of Dectin-1 can suppress M1 macrophage polarization and alleviate myocardial IRI (38). T cells can continue to localize in injured kidneys for 2 weeks and display an effector-memory and activation phenotype characterized by CD44^hi^CD62L^−^ and CD69^+^ expression, respectively (39). Treg function is decreased in aged mice, which contributes to exacerbated liver IRI (40).

## Hypoxic Stimuli

In transplantation, immune-mediated injury is a composite of the innate response to IRI and alloimmune reactivity to foreign Ag. Many clinical studies confirm a link between delayed graft function and a higher rate of acute rejection (41–44). We and others have shown that hypoxia activates DCs (45), as evidenced by their phenotypic maturation, pro-inflammatory cytokine production, and enhanced T cell stimulatory and migratory capacity (46–50).

Altered DCs function under hypoxia has been ascribed to changes in hypoxia-inducible factor (HIF)-1α activity. Longer-term hypoxia (>1 h) followed by reperfusion downregulates the G-protein coupled purinergic receptor P2Y11R through HIF-1α transcriptional regulation, resulting in augmented production of IL-12 and IL-6 and an inflammatory T cell phenotype in response to extracellular ATP (51). Hypoxia, and therefore HIF-1α-dependent upregulation of adenosine receptor A2B (52), or triggering receptor expressed on myeloid cells- (TREM)-1 (53, 54) can induce Th2 polarization and pro-inflammatory cytokine release, respectively. BMDCs lacking functional HIF-1α show deficiencies in type I IFN secretion and fail to activate CD8^+^ T cells (55). Interestingly, the effect of hypoxia on DCs is attenuated by rapamycin (47) and augmented by concurrent exposure to LPS (56).

Together, these data suggest that DCs are reprogrammed by a hypoxic environment to modulate their inflammation-activating repertoire, however discrepant features in many studies may reflect differences in hypoxia duration and severity. *In vivo*, HIF-1α is crucial for DC-dependent generation of Tregs and Treg homing to inflammatory sites. Absence of DC-specific HIF-1α limits IL-10 and TGF-β expression (both potent inducers of Tregs) and reduces expression of aldehyde dehydrogenase (necessary for catalytic production of retinoic acid) (57). Although HIF-1α is protective against IRI (58), there are no studies assessing its specific effect on DCs in this model.

## Ischemic Preconditioning

Ischemic preconditioning (IPC) occurs when brief periods of sublethal ischemia are performed prior to a subsequent prolonged episode and increases organ resistance to IRI (59). However, the underlying mechanism (s) remain elusive. Exposure of renal parenchyma to IPC prior to IRI does not alter the number of infiltrating leukocytes, but reduces CD11c^+^ DCs (compared to IRI alone), which display lower CCR7 and IL-17 transcript levels, decreased CD80 expression and upregulated IL-10 (60). Elimination of CD11c^+^ DCs (CD11b^+^ and CD8^+^ subsets) using liposomal clodronate is associated with partial loss of preconditioning benefits.

## Organ Cross-Talk During IRI

IRI rarely occurs in isolation—systemic release of pro-inflammatory cytokines and cell trafficking to primary and secondary lymphoid tissue ensures widespread modulation of innate immunity. Substantial cross-talk between the injured and remote organs manifests clinically as multi-organ failure. The onset of acute lung injury in the context of acute kidney injury (AKI) occurs more frequently than any other organ combination (61). Experimental models have identified a distinct pulmonary genomic signature during AKI, with differentially expressed pro-inflammatory and pro-apoptotic pathways (62). The intestine can also aggravate the systemic inflammatory response syndrome (63). Increased permeability from gut hypoperfusion, modification of microbiota composition, and blood-borne propagation of toxins contribute to the outcome of AKI. Gut microbiota can also modulate the inflammatory response to injury. Short-chain fatty acids such as acetate, propionate, and butyrate are produced by fermentation of complex carbohydrates and have a broad range of anti-inflammatory effects (64). Concurrent incubation of murine BMDCs with short-chain fatty acids and LPS reduces upregulation of maturation markers CD80, CD86, and CD40 (65). Butyrate specifically inhibits production of IL-12 in human monocyte-derived DCs, limiting development of effector Th1 cells (66, 67). Treatment with short-chain fatty acids also protects against AKI, with lower frequencies of infiltrating macrophages and activated DCs (65) in addition to effects on renal tubular epithelial cell apoptosis and ROS production.

## Organ-Specific Roles of DCs in IRI

### Tissue-Specific Phenomena

The majority of animal data on the function of DCs in IRI is limited to the liver, heart and kidney, and is best characterized in the latter. Robust models of pulmonary, intestinal and pancreatic IRI are lacking due to technical challenges. Human data is also sparse due to inherent difficulties with DC detection and limited tissue availability. *In vivo* targeting of organ-specific DCs to limit IRI and innate immune activation is difficult due to the lack of defining cell-surface markers. Tables 1, 2 outline the breadth of available markers, which show significant overlap.

**Table 2:** Identified DC subsets implicated in IRI.

**Organ**	**Mouse**	**Human**	**Function**
Heart (68)	**Steady-state** **Myeloid DC:** CD45^+^Lin^−^CD11c^+^MHCII^+^ **cDC1:** CD103^+^CD11b^−^, Clec9a, Flt3/CD135, CD205, CD24, CD283 **cDC2**: CD103^−^ CD11b^+^, CD115/M-CSF^lo^, F4/80^lo^, CX3CR1^lo^, Ly6C^lo^ **Double negative (DN) cDC**: CD103^−^CD11b^−^ **Plasmacytoid DC**:CD45^+^ Lin^−^ CD46^−^ MHCII^lo^ CD11b^−^ CD11c^lo^ PDCA1^+^ Ly6C^+^ SiglecH^+^ Clec9a^+^	**Steady-state** Hu-mice reconstituted with human stem cells + Flt3L: **Myeloid DC**: HLA-DR^+^ CD11c^+^ BDCA1^+^ (CD1c^+^), IRF4 BDCA3^+^, IRF8 **Plasmacytoid DC:** CD123^+^BDCA2^+^LAMP^+^IRF8	Murine: post-IRI 10-fold increase, DC depletion improves cardiac function post-MI (68), OR worsens LV function (69), increased DC worsen MI outcomes (70, 71), cDC2 increase numbers and CD40 expression in response to MI, prime autoreactive T cells, 4-fold increase, no functional role post-MI
Liver (72)	**mDC:** CD11c^+^CD8α^−^CD11b^+^ **CD8α^+^** **DC:** CD11c^+^ CD8α^+^CD11b^+^ GM-CSF administration: CD11c^+^CD11b^+^B220^−^CD205^−^ **pDC:** CD11c^lo^B220^+^Ly6C^+^CD11b^−^ **NK-DC:** CD11c^+^NK1.1^+^	Liver perfusate and explanted livers CD11c^+^ DC subsets (72): CD141^+^ Clec9A^+^ (30% of total CD11c population) ILT3^+^ (38%) ILT4^+^ (52%) CD1c^+^ (20% of total CD11c population) **Plasmacytoid DC:** HLA-DR^+^Lin^−^CD11c^−^CD123^+^ (15%)	Human: CD141^+^ cells enriched in healthy livers, secrete CXCL10, IL-1β, IL-17, and IFN-γ; initiate Th1/Th17 responses, express TLR3 Mouse: 65% reduction in cDC post-IRI (73), DC depletion (CD11c-DTR system) worsens IRI (73), Flt3L KO depletes DC and protects against IRI (74), CD11b^hi^ cells increase CD80/86 expression (33), CD40 (DC)-CD154 (T cell) ligation activates innate immunity (75)
Kidney	**Steady-state** **Mononuclear phagocyte subsets** (76): 1. CD11b^hi^CD11c^hi^: MHCII^+^ CCR2^+^ CD16^+^ Zbtb46^+^ 2. CD11b^hi^CD11c^lo^: CCR2^+^ CSF1R^+^ 3. CD11b^int^CD11c^int^: F4/80^+^CD14^+^CX3CR1^+^CSFR1^+^ MHCII^+^IL-10^+^ 4. CD11b^lo^CD11c^hi^: CD103^+^CCR7^+^ Zbtb46^+^Batf3^+^IRF8^+^ 5. CD11b^−^CD11c^int^ IRF8^+^ **Post-IRI** CD45^+^CD11c^+^ MHC-II^+^ TNF-α^+^CD80^+^CD86^+^CD40^+^ CD54^+^ (ICAM), C1d^+^ CD8α^−^CD4^−^CD205^−^ (77)	**Steady-state** (78, 79) **Myeloid DC**: Lin^−^HLA-DR^+^ **cDC1:** CD11c^+^ CD141^+^Clec9A^+^ **cDC2:** CD11c^+^CD1c^+^ CD1a^+^ (subset) **Plasmacytoid DC**: Lin^−^HLA-DR^+^CD11c^−^CD123^+^ CD303^+^	IRI increases: total CD45+ cells, CD45+CD11c–Ly6C+ (monocytes), CD45+CD11c–Ly6G+ (neutrophils), CD45+CD11c+ Ly6C–F4/80– (DC). CD45^+^CD11c^+^Ly6C^−^F4/80^+^ DC were unchanged (77). CD11c^+^ DC present Ag to T cells in draining renal lymph node (80).
Pulmonary (81, 82)	**Steady-state** **Myeloid DC:** CD11c^hi^ **Airways:** CD103^+^CD11b^lo^CD207^+^ (Langerin), XCR1^+^Clec9A^+^ Batf3, ID2, IRF8, Zbtb46 **Beneath basement membrane:** CD103^−^CD11b^+^ RELB, IRF2, IRF4, Zbtb46 **Plasmacytoid DC:** CD11c^int^ MHC II^int^B220^+^Ly6C/Gr-1^hi^Siglec-H^+^BST-2^+^ IRF8, E2-2	**Steady state** **Myeloid DC:** HLA-DR^+^ CD1c^+^CD11c^+^ CD14^−^ **Plasmacytoid DC:** HLA-DR^+^ CD123^+^ CD11c^−^CD14^−^ **Maturation:** CD83^+^	Tightly associated with conducting airways and epithelia (83). No information in IRI models.
Intestine (84, 85)	**Steady state** **cDC1:** CD103^+^ XCR1^+^ CD11b^−^CD172^−^ IRF8, Batf3 **cDC2:** CD103^+^CD11b^+^CD172^+^ IRF4, Notch2 or_CD103^−^CD11b^+^CX3CR1^int^	**Steady state** **cDC1:** CD103^+^CD141^+^ XCR1^+^DNGR1^+^ **cDC2:** CD103^+^CD172^+^ CD141^−^ orCD103^−^ CD172^+^CD141^−^	cDC1 and cDC2 CD103^+^ cells within epithelium, lamina propria, and draining lymph nodes

*Batf, basic leucine zipper transcriptional factor-ATF like; BDCA, blood dendritic cell antigen; Clec, C-type lectin receptor; CSF, colony stimulating factor; FLT3, fms like tyrosine kinase 3; HLA, human leukocyte antigen; MHC, major histocompatibility complex; ICAM, intercellular adhesion molecule; ID, inhibitor of DNA binding; IFN, interferon; IL, interleukin; ILT, immunoglobulin-like transcript; IRF, interferon regulatory factor; IRI, ischemia reperfusion injury; Lin, lineage; MI, myocardial infarction; RELB, v-rel avian reticuloendotheliosis viral oncogene; Siglec, sialic acid binding immunoglobulin like lectin; Zbtb, zinc finger and BTB domain-containing protein*.

### Kidney

CD11c^+^ DCs are resident within the kidney parenchyma (86), infiltrate following ischemic insult and are the dominant TNFα-producing cells (77) (Figure 1). TNF-α is crucial to neutrophil influx post-IRI, renal tubular epithelial cell apoptosis, glomerular endothelial damage and fibrin deposition (87–90). The effect of TNF-α depends on the transcription factor IRF-4 (91). Non-specific elimination of DC using liposomal clodronate or administration of etanercept (a decoy receptor for TNF-α) abrogates AKI in IRF-4 deficient mice (91).

Intra-renal DCs post-IRI constitutively express markers that designate them as professional APCs (MHC, CD80, CD86, CD40, CD54, CD1d), but not tissue macrophage markers [CD169, CD204 (77)]. As early as 4 h post-IRI, higher levels of maturation marker expression are observed, favoring the hypothesis that DC maturation occurs *in situ* rather than by cell replacement (77). Multiple studies have identified a continuum of DC phenotypes that contribute to the innate immune response and IRI. Monocyte subsets migrate to inflamed tissue and differentiate into activated DCs as CCR2^+^CX3CR1^lo^GR-1^+^Ly6C^hi^ cells (92). Resident CX3CR1^hi^ monocytes patrolling the parenchymal space also migrate, differentiate, and participate in inflammation. Both CCR2 (93) and CX3CR1 (94) are essential to this process.

The traditional paradigm has been discrete separation of mononuclear phagocytic cell populations into CD11b^+^ macrophages and CD11c^+^ DCs that increase following IRI. More recent thinking has focused on phenotypic heterogeneity and functional plasticity of these cells. They are divided broadly into 5 subsets based on intensity of CD11b and CD11c expression and are further characterized by a comprehensive set of cell surface markers and transcription factors (76). All subsets demonstrate phagocytic capacity but differ in their migratory capacity and cytokine profile as well as their ability to stimulate naïve T cells and alter T cell polarization *ex vivo*. Renal DCs are capable of presenting self-Ag to a variety of T cells in the context of IRI, -CD11c^+^ DCs cross-present Ag to CD8^+^ T cells post-IRI (95) and glycolipids are presented to NKT cells via the CD1d cell surface receptor (96). Activation of NKT via CD40 initiates IFN-γ to amplify the innate immune response. Administration of CD1d Ab that blocks NKT-DC interactions or genetic depletion of NKT provides significant protection against renal IRI (96). Renal DCs are also primarily responsible for presenting renal proteins to Ag-specific CD4^+^ T cells within draining renal lymph nodes. In a model of unilateral IRI where ovalbumin is placed beneath the operated kidney capsule, CD11c^+^ DCs from the ipsi- or contra-lateral renal lymph nodes induce proliferation of DO11.10 (ovalbumin-restricted) T cells (80). CD11c^−^ fractions failed to induce T cell stimulation.

Naïve rodent kidneys demonstrate additional DC subsets defined by the expression (or absence) of CD103^+^. The CD103^+^ subset is primarily involved in Ag cross-presentation after migrating to lymph nodes (97). Transplantation of syngeneic grafts subjected to negligible or prolonged cold storage leads to depletion of CD103^+^ DCs, regardless of IRI, but only CD103^−^ DCs under the former condition (98). Donor cells are replaced by host DCs, accompanied by increases in CD3^+^CD4^+^CD62L^−^ T cells, indicative of effector/effector-memory populations.

### Heart

Despite advances in percutaneous coronary intervention and use of statin and antiplatelet agents, the incidence of post-infarct heart failure is rising (99). Adverse ventricular remodeling following myocardial infarction increases mortality by precipitating heart failure. Studies of DC subsets in experimental myocardial IRI are relatively uncommon, mostly due to the technical difficulty, high mortality, and significant variations in infarct size associated with left anterior descending artery ligation (100, 101).

DC subsets are found within the CD45^+^ leukocyte population in healthy myocardium, particularly within the right atrium (68). Conventional CD11c^+^MHC II^+^ DCs (cDC1s) have been defined by CD103 or CD11b expression (or neither) (68). These 2 subsets are also classified by XCR1 or CD172 (SIRP-α) expression, and levels of transcription factors IRF8 and IRF4, respectively (102). Under homeostatic conditions, cDC1s present cardiac self-Ag to α myosin heavy chain-specific CD4^+^ T cells in mediastinal lymph nodes to induce Treg formation. pDCs (CD11b^lo^MHCII^−^CD11c^lo^PDCA-1^+^Ly6C^+^) have also been detected.

DCs infiltrate infarcted myocardium (103), increasing 10-fold, particularly the CD11b^+^ subset expressing maturation markers such as CD40 (68). Post-myocardial infarction cDCs upregulate CCR7 (the chemokine receptor required for lymph node migration) and CD40 expression; specific activation of the cDC2 subset induces CD86 expression as well as CD4^+^ T cell proliferation and accompanies IFN-γ and IL-17 production (102). Although mature DC numbers correlate with LV dysfunction (70) and depletion of cDCs reduces infarct size and adverse cardiomyocyte hypertrophy in the border zone (68), a deleterious contribution of DCs is disputed. Prolonged DC ablation leads to impaired LV remodeling, sustained expression of pro-inflammatory cytokines and altered monocyte/macrophage recruitment (69). The administration of liposomal clodronate, which depletes both DCs and macrophages, also impairs wound healing (104). pDC numbers also increase following infarction, but their depletion appears not to affect LV function (68).

DCregs have therapeutic potential in post-infarct healing, modulating Tregs and macrophages. “Tolerogenic DCs” primed with TNF-α and mouse cardiac tissue Ags reduced infarct size, improved LV ejection fraction and increased post-infarct survival (105). This correlated with increased FoxP3+ Tregs in inguinal and mediastinal lymph nodes, as well as in the post-infarct heart. Adoptive transfer of these Tregs into mice post-MI also improved wound healing. Interestingly, troponin- or myosin-primed DCs failed to recapitulate the protective effects seen with tissue-primed DCs.

### Liver

Similar roles for DCs have been demonstrated in hepatic IRI. Interestingly, cDCs are depleted from the liver parenchyma, rather than enriched, during maximal injury (12 h post-reperfusion). These DCs display a mature phenotype, with marked CD86 upregulation, but are also necrotic and apoptotic. Depletion of CD11c^+^ DCs in CD11c-DTR mice worsens tissue damage and the pro-inflammatory cytokine profile following liver IRI. Adoptive transfer of cDCs into CD11c-depleted mice mitigates this injury, dependent on intrinsic TLR9 activation (from hepatocyte DNA release) and subsequent IL-10 production (73). This is also thought to modulate CD11b^int^Ly6C^hi^ inflammatory monocyte cytokine production and ROS generation. Augmenting DC numbers with GM-CSF increases susceptibility to liver IRI (106). Post-reperfusion, these DCs exhibited a mature phenotype and enhanced allostimulatory capacity. This effect required release of high motility box group 1 that upregulates DC-expressed TLR4 and interacts with both TLR9 and the receptor for advanced glyosylation end-products (RAGE) to activate DCs (107).

Liver transplant recipients are more likely to develop a tolerant phenotype compared to recipients of other solid organs. This may be due to a comparatively high density of DCs to parenchymal cell populations (108) and the refractory behavior of liver DCs compared with DCs from other tissues in response to TLR ligation (109, 110). Expression of CD39, which drives ATP hydrolysis, is increased on murine liver mDCs but not pDCs, and the levels exceed those in splenic DCs. Not unexpectedly, CD39KO syngeneic liver transplants exhibit worse tissue injury compared to WT grafts, with accompanying upregulation of CD80, CD86, and MHC II, and downregulation of PD-L1 on hepatic mDCs (109). Notably, CD39 expression on freshly isolated human hepatic CD45^+^Lin^−^BDCA-1^+^ DCs is higher than on peripheral blood mDCs.

The discrepant findings suggesting a dichotomous role for DCs in liver IRI imply that tissue-resident and recruited DCs may play distinct roles in the response to injury. DCs are highly motile, and are recruited into the liver in response to macrophage inflammatory peptide-1 (111) (MCP-1 = CCL2), which is produced following IRI. Administration of Flt3L to WT mice increases the DC-resident population 10-fold (112) and enhances liver parenchymal injury; injury is significantly reduced in Flt3L KO mice (74). Adoptive transfer of CD11c^+^ mDCs or PDCA-1^+^ pDCs into Flt3L KO mice at the time of IRI significantly worsens injury. To distinguish the contribution of hepatic-resident vs. infiltrating DCs, a liver transplant model between congenic mice has been used. Donor-derived hepatic DCs upregulated maturation markers to a higher level than on infiltrating (recipient) DCs. Use of Flt3L KO liver donors increased ischemic injury, suggesting a protective effect of liver-resident DCs.

The role of pDCs in liver IRI is poorly defined. IFN-α is produced predominantly by pDCs (113) in response to sensing of self-DNA and TLR9 ligation, which drive the inflammatory response in IRI. IFN-α promotes IRF-1 expression by hepatocytes to induce pro-inflammatory cytokine and death receptor expression (34). Depletion of pDCs using anti-PDCA-1 Ab or use of IFN-α blocking Ab protected the liver against IRI.

### The Therapeutic Potential of DC

Despite decades of research and multiple clinical trials, no pharmacological agents are in clinical use for IRI. Cell-based therapy, which capitalizes on the capacity of regulatory cells to modulate adverse immune responses, represents a potential therapeutic option. DCs can exhibit a protective phenotype to modulate pathogenesis of IRI. However, unlike adoptive transfer of Tregs, which can change to a proinflammatory/effector phenotype, depending upon the microenvironment (114, 115), DCregs are maturation-resistant, phenotypically stable and do not need to persist *in vivo* as functional cells to mediate immune response change (116). Pharmacological or genetic manipulation produces a regulatory phenotype (DCregs), secreting low levels of Th1-driving cytokines (particularly bioactive IL-12), and comparatively high levels of anti-inflammatory cytokines (such as IL-10) (117). They are weak T cell stimulators and possess the capacity to induce or expand Tregs. While the beneficial effect of DCregs has been established in multiple pre-clinical models of allograft rejection [reviewed in Thomson et al. (118)], their testing in IRI has been minimal. Elegant experimental work has demonstrated that following adoptive transfer, allogeneic DCregs are rapidly killed by host immune cells (predominantly NK cells), and their effect is mediated by recipient DCs (119, 120). Even in the absence of alloAg, adoptively transferred DCs can direct T cells to produce a regulatory response and mitigate IRI. Thus, while lack of the sphingosine-1-phosphate receptor 3 (S1pr3) protects against renal IRI (121), infusion of S1pr3^−/−^ DCs also protects against IRI which requires functional Tregs, CD11c^+^ DCs, and CD169^+^ macrophages (122). In addition, DCs treated with an adenosine 2A receptor agonist protect WT mice from IRI via suppressing NKT cell activation and IFN-γ production (123).

### Current Gaps in Knowledge

Variations and limitations in methodology, both for identification and *ex vivo* generation of DCs, have made it difficult to determine whether phenotype and function are consistent between organs affected by IRI. Which self-Ags DCs respond to in IRI is not well-defined and clearly differs according to damaged organ segment, cell type or cellular component (glomerulus vs. tubule, hepatocyte vs. endothelia, myosin heavy chain vs. tubulin). In addition, there is still no consensus to clearly distinguish tissue DCs and macrophages (124, 125). Cell-surface molecules such as CD11c and CX3CR1 are expressed on various myeloid cells and are imperfect DC markers (126). A further caveat is that *ex vivo*-generated DCs do not truly represent a physiological subset of tissue-resident DCs. The majority of studies to date have been conducted in animal models of IRI, and the paucity of human data highlights limitations regarding generalizability of results.

DCregs are currently being tested as cell therapy in clinical liver and kidney transplantation, and the results of these trials are eagerly awaited. If DCregs are to be considered as therapy, it will be necessary to determine cell type (autologous vs. allogeneic vs. donor Ag-loaded), dosage, timing, and frequency of infusion (s) and cost. Other aspects, such as potential sensitization of recipients (with the use of banked allogeneic DCs) and comparison of DCregs with other regulatory cell types, have also not been addressed. It is also unclear whether the relative efficacy of DC therapy also depends on the organ undergoing IRI. Published research has also focused on the effect of DCs given prior to the onset of IRI, whereas their influence post-injury is not known.

## Author Contributions

All authors listed have made a substantial, direct and intellectual contribution to the work, and approved it for publication.

### Conflict of Interest

The authors declare that the research was conducted in the absence of any commercial or financial relationships that could be construed as a potential conflict of interest.
